# Longitudinal Trajectories and Factors Associated With US County-Level Cardiovascular Mortality, 1980 to 2014

**DOI:** 10.1001/jamanetworkopen.2021.36022

**Published:** 2021-11-30

**Authors:** Shreya Rao, Amy Hughes, Matthew W. Segar, Brianna Wilson, Colby Ayers, Sandeep Das, Ethan A. Halm, Ambarish Pandey

**Affiliations:** 1 Division of Cardiology, Department of Internal Medicine, University of Texas Southwestern Medical Center, Dallas; 2 Department of Population and Data Sciences, University of Texas Southwestern Medical Center, Dallas; 3 School of Medicine, University of Texas Southwestern Medical Center, Dallas; 4 Department of Internal Medicine, University of Texas Southwestern Medical Center, Dallas

## Abstract

**Importance:**

Cardiovascular (CV) mortality has declined for more than 3 decades in the US. However, differences in declines among residents at a US county level are not well characterized.

**Objective:**

To identify unique county-level trajectories of CV mortality in the US during a 35-year study period and explore county-level factors that are associated with CV mortality trajectories.

**Design, Setting, and Participants:**

This longitudinal cross-sectional analysis of CV mortality trends used data from 3133 US counties from 1980 to 2014. County-level demographic, socioeconomic, environmental, and health-related risk factors were compiled. Data were analyzed from December 2019 to September 2021.

**Exposures:**

County-level characteristics, collected from 5 county-level data sets.

**Main Outcomes and Measures:**

Cardiovascular mortality data were obtained for 3133 US counties from 1980 to 2014 using age-standardized county-level mortality rates from the Global Burden of Disease study. The longitudinal K-means approach was used to identify 3 distinct clusters based on underlying mortality trajectory. Multinomial logistic regression models were constructed to evaluate associations between county characteristics and cluster membership.

**Results:**

Among 3133 US counties (median, 49.5% [IQR, 48.9%-50.5%] men; 30.7% [IQR, 27.1%-34.4%] older than 55 years; 9.9% [IQR, 4.5%-22.7%] racial minority group [individuals self-identifying as Black or African American, American Indian or Alaska Native, Asian, Native Hawaiian, Pacific Islander, other, or multiple races/ethnicities]), CV mortality declined by 45.5% overall and by 38.4% in high-mortality strata (694 counties), by 45.0% in intermediate-mortality strata (1382 counties), and by 48.3% in low-mortality strata (1057 counties). Counties with the highest mortality in 1980 continued to demonstrate the highest mortality in 2014. Trajectory groups were regionally distributed, with high-mortality trajectory counties focused in the South and in portions of Appalachia. Low- vs high-mortality groups varied significantly in demographic (racial minority group proportion, 7.6% [IQR, 4.1%-14.5%]) vs 23.9% [IQR, 6.5%-40.8%]) and socioeconomic characteristics such as high-school education (9.4% [IQR, 7.3%-12.6%] vs 20.1% [IQR, 16.1%-23.2%]), poverty rates (11.4% [IQR, 8.8%-14.6%] vs 20.6% [IQR, 17.1%-24.4%]), and violent crime rates (161.5 [IQR, 89.0-262.4] vs 272.8 [IQR, 155.3-431.3] per 100 000 population). In multinomial logistic regression, a model incorporating demographic, socioeconomic, environmental, and health characteristics accounted for 60% of the variance in the CV mortality trajectory (*R*^2^ = 0.60). Sociodemographic factors such as racial minority group proportion (odds ratio [OR], 1.70 [95% CI, 1.35-2.14]) and educational attainment (OR, 6.17 [95% CI, 4.55-8.36]) and health behaviors such as smoking (OR for high vs low, 2.04 [95% CI, 1.58-2.64]) and physical inactivity (OR, 3.74 [95% CI, 2.83-4.93]) were associated with the high-mortality trajectory.

**Conclusions and Relevance:**

Cardiovascular mortality declined in all subgroups during the 35-year study period; however, disparities remained unchanged during that time. Disparate trajectories were associated with social and behavioral risks. Health policy efforts across multiple domains, including structural and public health targets, may be needed to reduce existing county-level cardiovascular mortality disparities.

## Key Points

**Question:**

How have trajectories of cardiovascular (CV) mortality among US counties varied over 35 years, and are county-level factors associated with this variation?

**Findings:**

In this cross-sectional study, cardiovascular mortality declined in all groups from 1980 to 2014 in this cross-sectional analysis of 3133 US counties. Three unique phenogroups based on mortality trajectory were identified, with the difference between high- and low-mortality counties unchanged during the study period, and were associated with social, behavioral, and environmental characteristics at the county level.

**Meaning:**

These findings suggest that despite an overall decline in CV mortality rates over the past 35 years, differences in health behavior patterns and other societal risk factors are associated with persistent CV mortality disparities at the county level.

## Teaser

This cross-sectional study identifies clusters based on cardiovascular mortality trajectory among US counties and county-level social, demographic, environmental, and health-related risk factors that are associated with these clusters.

## Introduction

Since the mid-20th century, cardiovascular (CV) disease has remained the leading cause of death in the US, peaking in the 1960s and subsequently declining owing to improvements in traditional CV risk factors and therapeutic advancements.^1,2^ The early 21st century has seen these declines slowing nationally with the prevalence of CV disease, driven by a growing epidemic of obesity, type 2 diabetes, and physical inactivity, now expected to rise by 10% in the coming decade.^3,4^ However, gains have been unequally distributed, with disparities in absolute CV mortality observed by geographic region, sex, and race and ethnicity.^5,6^

Despite a large body of literature establishing their impact on CV health, social and environmental risk factors in the US have been excluded from national studies of CV mortality.^4^ This exclusion is due in large part to challenges in defining and measuring social indicators. Each of the 3 most commonly reported socioeconomic measures—educational attainment, income, and occupation—is associated with the prevalence of CV risk factors and CV mortality risk.^4,7,8^ Disparities in socioeconomic factors have expanded in recent decades, potentially contributing to the rise in CV disease observed in this period.^9,10,11^ However, health-related risk factors have remained the primary targets for intervention nationally.^3^

Studies of CV mortality to date have been limited to cross-sectional assessments with inadequate capture of social, demographic, and environmental risk factors for CV mortality at the county level. Although prior studies have demonstrated a substantial decline in CV mortality in the past few decades, county-level heterogeneity in temporal trends has not been well studied.^5,6,12,13^ Counties represent one of the smallest geographic units for which valid multisectoral health outcome and behavioral data are available and meaningful in the US, because they represent adequate sample sizes for aggregation while remaining relevant for health policy implementation. Thus, understanding geographic variation in CV mortality trends over time and its drivers at the county level provides a unique opportunity for identifying actionable social and health policy targets for improving CV outcomes. In this study, we identify clusters based on CV mortality trajectory from 1980 to 2014 among US counties and county-level social, demographic, environmental, and health-related risk factors that are associated with CV mortality trajectory-based clusters.

## Methods

### Study Overview

In this cross-sectional longitudinal study, we evaluated trajectories in annual CV mortality for 3133 US counties for which complete data were available for the 35 years from 1980 to 2014 in the Global Burden of Disease (GBD) program. Ten counties were excluded from analysis owing to missing data. Deaths were tabulated by county for each year in the follow-up period. County-level characteristics were compiled from 5 independent data sources to capture demographic, socioeconomic, environmental, and health-related risk factors for all counties included in the analysis. Detailed descriptions of these data sources are provided in eMethods in the Supplement. Race was self-reported by respondents of the American community survey and was included owing to the established racial differences in cardiovascular mortality and other societal and social determinants of health. This study was performed with publicly available deidentified data and received institutional review board exemption from the University of Texas Southwestern Medical Center, Dallas. The present study is reported in accordance with the Strengthening the Reporting of Observational Studies in Epidemiology (STROBE) reporting guideline.^14^

### County-Level CV Mortality Rate

For this analysis, we obtained yearly county-level age-standardized CV mortality data from 1980 to 2014 reported as deaths per 10 000 person-years from the Institute for Health Metrics and Evaluation’s GBD program. Briefly, the GBD study aggregates deidentified death certificate data obtained from the National Center for Health Statistics for each US state and population counts obtained from the US Census Bureau.^5,6,15,16^ Cause of death by death certificate was determined by codes from the *International Classification of Diseases, Ninth Revision,* through 1999, and *International Statistical Classification of Diseases and Related Health Problems, Tenth Revision,* after 1999. Deaths attributable to ischemic heart disease, ischemic stroke, hemorrhagic and other stroke, atrial fibrillation, peripheral artery disease, aortic aneurysm, cardiomyopathy and myocarditis, hypertensive heart disease, and rheumatic heart disease were combined to yield total deaths attributable to CV disease. The GBD study estimates age-standardized county-specific CV death rates using small-area estimation methods.^6^

### County-Level Characteristics

We obtained county-level characteristics from 5 independent data sources encompassing 4 categories: population demographics, socioeconomic data, physical environment, and health status. Population characteristics and sociodemographic data were measured using 5-year estimates for 2011 to 2015 from the American Community Survey,^17^ including measures of counties’ racial and ethnic composition, sex distribution, the proportion of the population older than 55 years, income, educational attainment, and violent crime rates.^18^ Economic distress, reported as housing vacancy rate, the proportion of adults not in work, the proportion of the population in a known distressed zip code, and aggregate distress score, was reported based on data collected from the Distressed Communities Index (DCI), developed from 2014-2018 American Community Survey data.^19^ Population distribution was determined from the US Department of Agriculture Economic Research Service Rural-Urban Continuum Codes.^17,20^ The health status of the population was measured using data reported in the 2014 County Health Rankings data set,^21^ which captures county-level data on the prevalence of health outcomes, risk factors, and physical environment. A summary measure of the food environment in each county and the percentage of tracts within a county classified as a food desert in 2010 was obtained from the US Department of Agriculture Food Environment Atlas.^22^ A list of included covariates and data sources is provided in eTable 1 in the Supplement.

### Statistical Analyses

Data were analyzed from December 2019 to September 2021. County-level CV mortality trajectories were modeled using the longitudinal K-means approach (R package KmL [R Program for Statistical Computing version 3.6.3]) to identify subgroups of counties with distinct mortality trajectories during the study period.^23^ Briefly, the longitudinal K-means approach partitions data into similar clusters using an unsupervised machine-learning algorithm. We determined the optimal number of clusters according to the elbow method; that is, the optimal number of clusters was the highest number of clusters before the marginal gain of additional clusters decreased.^24^ Each county was assigned to a cluster with optimal clustering using expectation-maximization methods wherein the centers of different clusters are computed during an expectation phase, and each observation is assigned to its nearest cluster in a maximization phase. Both phases are repeatedly alternated until no further change occurs in cluster assignments. County-level characteristics across the CV mortality trajectory-based clusters were reported as medians (IQR) and compared using 1-way analysis of variance. Generalized linear mixed-effect models were constructed to evaluate the rate of change of mortality by trajectory subgroup. Geographic clustering of the US counties based on their CV mortality trajectory was assessed visually by choropleth. A choropleth of counties based on the proportion of the population living in a distressed zip code was constructed separately using economic distress data from the DCI.

We constructed hierarchical multinomial logistic regression models to evaluate the independent associations of various county-level characteristics with trajectory cluster membership. Collinearity among covariates was assessed, and if 2 variables had a correlation coefficient of greater than 0.7, the variable believed to represent the most upstream factor in the risk pathway was retained in the model. We fitted 4 nested models by sequentially adding groups of county-level characteristics. Model 1 included demographic characteristics (population percentage of individuals from racial minority groups [including individuals self-identifying as Black or African American, American Indian or Alaska Native, Asian, Native Hawaiian, Pacific Islander, other, or multiple races/ethnicities], Hispanic, male individuals); model 2, model 1 plus socioeconomic characteristics (percentage with less than a high-school education and median household income); model 3, model 2 plus community structural attributes (proportion of food deserts, violent crime rates, access to exercise opportunities, and housing vacancy rates); and model 4, model 3 plus health and behavioral risk factor burden (proportion of the population with a body mass index [calculated as weight in kilograms divided by height in meters squared] of greater than 30, smoking prevalence, and percentage of adults with no leisure-time physical activity). Adjusted associations between county-level characteristics and the likelihood of CV trajectory were reported as odds ratios (ORs) with 95% CIs. Proportional variance in cluster membership was assessed using *R*^2^ values. Analyses were performed using SAS, version 9.4 (SAS Institute Inc), and R, version 3.6.3 (R Program for Statistical Computing) with a 2-sided *P* < .05 considered statistically significant.

## Results

**Figure 1.**
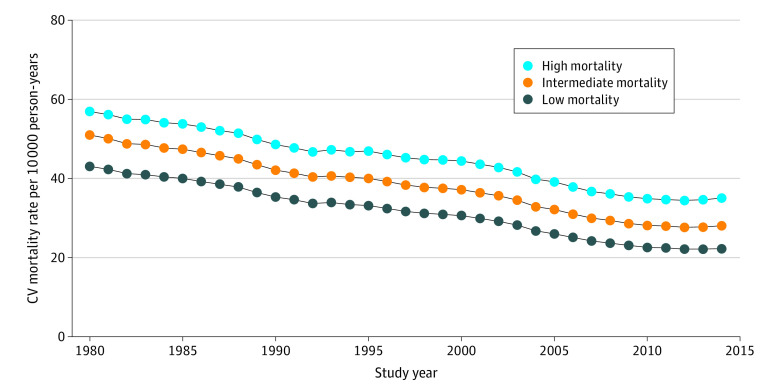
Cardiovascular (CV) Mortality Trajectory by Cluster, 1980 to 2014 Declines in CV mortality were observed in all clusters with the differences in absolute mortality rate between high- and low-mortality clusters unchanged during the 35-year study period. Mortality rates are reported as medians.

We evaluated annual CV mortality among residents of 3133 US counties (median, 49.5% [IQR, 48.9%-50.5%] men; 30.7% [IQR, 27.1%-34.4%] older than 55 years; 9.9% [IQR, 4.5%-22.7%] racial minority groups) from 1980 to 2014. Over the 35 years analyzed, mortality declined by 45.5% overall in the US (49.9 [IQR, 45.0-54.4] to 27.2 [IQR, 23.5-31.6] per 10 000 person-years). Three distinct county clusters were identified based on the trajectories in CV mortality, with mortality declining by 38.4% in high-mortality strata, by 45.0% in intermediate-mortality strata, and by 48.3% in low-mortality strata (Figure 1). Overall, CV mortality declined concomitantly in each county cluster during the 35-year study period; 1057 counties (34%) had low mortality rates throughout the study period (low-mortality cluster, 43.1 [IQR, 40.6-45.6] vs 22.3 [IQR, 20.5-24.3] per 10 000 person-years in 1980 and 2014, respectively), 1382 (44%) had intermediate mortality rates (intermediate-mortality cluster, 51.0 [IQR, 48.4-53.6] vs 28.0 [IQR, 26.0-30.4] per 10 000 person-years in 1980 and 2014, respectively), and 694 (22%) had high mortality rates (high-mortality cluster, 56.9 [IQR, 54.2-60.3] vs 35.1 [IQR, 32.5-38.0] per 10 000 person-years in 1980 and 2014, respectively). Notably, as each cluster experienced a similar absolute decline in CV mortality, the mortality gap between high-mortality and low-mortality counties persisted during the 35-year study period (13.8 vs 12.8 per 10 000 person-years in 1980 vs 2014, respectively), with trajectories remaining parallel among clusters. The rate of change in CV mortality over time was comparable for the low-, intermediate-, and high-mortality clusters for most of the study period (1980-2009) (eTable 2 in the Supplement). All clusters experienced a plateau in mortality rates from 2010 to 2014, with mortality rates increasing marginally among the high-mortality counties during this time.

### CV Mortality Trajectories and Regional Distribution

**Figure 2.**
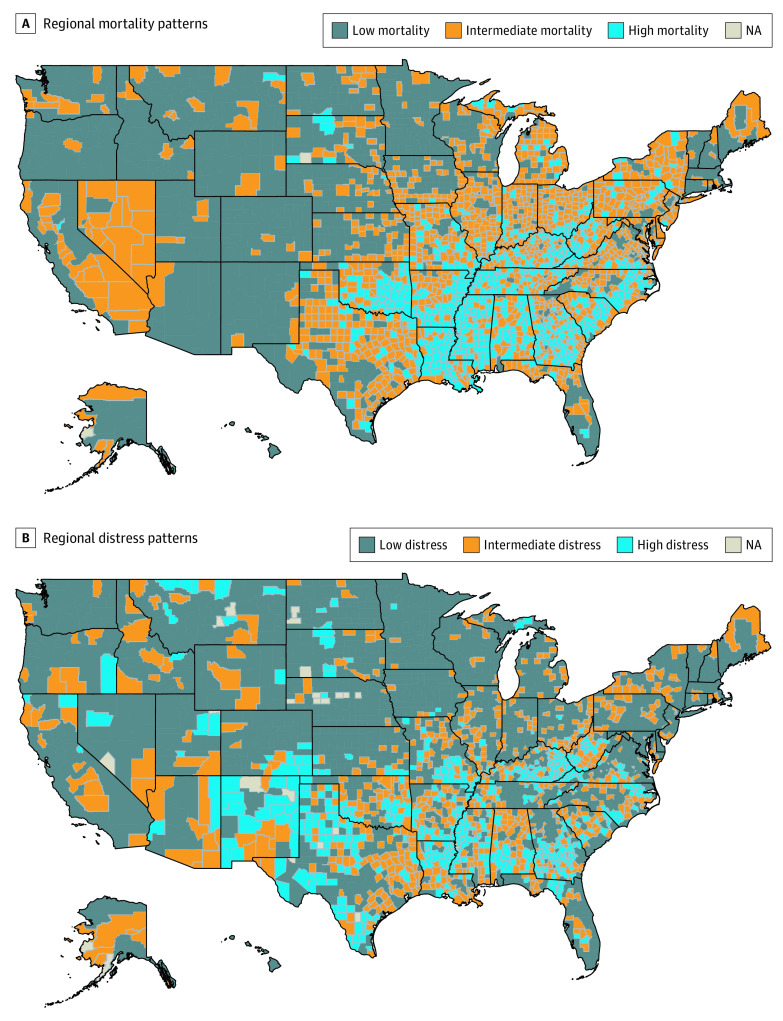
Choropleths Depicting Regional Patterns of Mortality and Distress A, Choropleth depicting regional distribution of cluster membership, with the highest-mortality counties clustered in the Deep South and Appalachia and the lowest-mortality counties clustered predominantly in the Northwestern, Western, and Midwestern US. B, Choropleth demonstrating the proportion of counties with greater than 80% population in distressed zip codes (highest distress), 20% to 80% population in distressed zip codes (intermediate distress), and less than 20% population in distressed zip codes (lowest distress). Substantial regional overlap is observed between counties with a high proportion of the population living in distressed zip codes and the high-mortality cluster.

Distinct regional patterns in county clusters were observed, with the most prominent separation among the high- and low-mortality clusters (Figure 2A). Low-mortality counties predominantly represented micropolitan, low-commuting counties in the Northwest, Mountain West, Great Plains, Southwest, Heartland, Southern Florida, and Northeast (Figure 2A). The intermediate-mortality cluster was the largest and consisted of micropolitan high-commuting counties on the West coast, in large portions of Nevada and Texas, and in portions of the Midwest/Plains Rust Belt, Northeast, New England, and South Atlantic regions. Finally, the high-mortality trajectory cluster represented the smallest grouping and was most regionally grouped in the Deep South, Appalachia, and South Atlantic regions. Substantial overlap was additionally observed between counties in the highest-mortality cluster and counties reporting a high burden of residents in socioeconomically distressed zip codes (Figure 2B).

### Characteristics of County Clusters on Mortality Trajectories

Descriptive characteristics of CV mortality–based county clusters are presented in Table 1. Age and sex distributions were similar across the 3 clusters; however, comparing high- vs low-mortality trajectory clusters, we observed a higher proportion of racial minority group residents (23.9% [IQR, 6.5%-40.8%] vs 7.6% [IQR, 4.1%-14.5%]), lower median household income ($36 900 [IQR, $33 300-$41 500] vs $51 000 [IQR, $44 800-$58 800]), a higher proportion of residents with less than a high school education (20.1% [IQR, 16.1%-23.2%] vs 9.4% [IQR, 7.3%-12.6%]), higher poverty rates (20.6% [IQR, 17.1%-24.4%] vs 11.4% [IQR, 8.8%-14.6%]), higher unemployment rates (31.3% [IQR, 27.5%-36.9%] vs 19.0% [IQR, 15.1%-24.0%]), higher uninsured rates (23.7% [IQR, 20.5%-26.4%] vs 18.6% [IQR, 13.7%-24.6%]), and higher violent crime rates (272.8 [IQR, 155.3-431.3] vs 161.5 [IQR, 89.0-262.4]) per 100 000 population. There were also significant differences in health and environmental characteristics across the CV mortality–based county clusters. Compared with low-mortality trajectory counties, those with a high-mortality trajectory included a greater proportion of food deserts (22.2% [IQR, 0.0%-35.7%] vs 6.8% [IQR, 0.0%-22.0%]), higher housing vacancy rates (13.7% [IQR, 11.3%-16.1%] vs 8.3% [IQR, 5.9%-12.6%]), higher rates of CV risk factors such as type 2 diabetes (12.9% [IQR, 11.7%-14.3%] vs 9.6% [IQR, 8.6%-10.9%]) and obesity (34.4% [IQR, 32.0%-36.9%] vs 28.6% [IQR, 25.5%-31.3%]), and risk behaviors such as smoking (21.8% [IQR, 19.6%-23.9%] vs 15.8% [IQR, 14.6%-17.1%]) and physical inactivity (32.2% [IQR, 29.6%-35.1%] vs 23.8% [IQR, 19.9%-27.3%]).

**Table 1.** Sociodemographic, Population, Health Status, and Food Environment Characteristics of US Counties by Mortality Trajectory Cluster

Characteristic	Overall cohort (3133 counties), median (IQR)	Mortality group, median (IQR)			*P* value for trend
		Low (1057 counties [34%])	Intermediate (1382 counties [44%])	High (694 counties [22%])	
**Demographic data**					
Population density, persons per square mile	45.1 (17.0-116.0)	25.1 (5.4-104.3)	60.4 (22.7-148.0)	45.1 (27.6-80.6)	<.001
Sex, %					
Female	50.5 (49.5-51.1)	50.1 (49.2-50.9)	50.5 (49.7-51.1)	50.8 (49.8-51.6)	<.001
Male	49.5 (48.9-50.5)	49.9 (49.1-50.8)	49.5 (48.9-50.3)	49.2 (48.4-50.2)	<.001
Age >55 y, %	30.7 (27.1-34.4)	31.8 (26.5-37.0)	30.7 (27.3-34.1)	29.8 (27.5-32.4)	<.001
Race and ethnicity, %					
Hispanic	3.7 (1.9-9.0)	5.4 (2.5-13.7)	3.6 (1.8-8.4)	2.5 (1.4-4.8)	<.001
Racial minority group^a^	9.9 (4.5-22.7)	7.6 (4.1-14.5)	9.7 (4.5-20.5)	23.9 (6.5-40.8)	<.001
White	90.1 (77.3-95.5)	92.4 (85.5-95.9)	90.3 (79.5-95.5)	76.1 (59.2- 93.5)	<.001
Population born outside the US, %	2.7 (1.4-5.7)	3.8 (1.9-8.4)	2.8 (1.4-5.5)	1.6 (0.9-3.0)	<.001
**Socioeconomic metrics**					
Distress score^b^	50.1 (25.0-75.0)	28.4 (12.6-49.2)	50.2 (28.7-70.5)	80.6 (65-91.4)	<.001
Population in distressed zip code, %	8.0 (0-58.0)	0 (0-6.0)	11.0 (0-50.0)	67.0 (30.0-95.8)	<.001
Median household income, $	45 200 (38 900-52 500)	51 000 (44 800-58 800)	45 600 (40 400-52 000)	36 900 (33 300-41 500)	<.001
Poverty rate, %	14.8 (11.0-19.1)	11.4 (8.8-14.6)	14.8 (11.6-17.6)	20.6 (17.1-24.4)	<.001
Less than high school education	13.1 (9.5-18.7)	9.4 (7.3-12.6)	13.3 (10.4-17.6)	20.1 (16.1-23.2)	<.001
Adults not in work, %	24.0 (18.9-30.5)	19.0 (15.1-24.0)	23.8 (20.1-29.1)	31.3 (27.5-36.9)	<.001
Uninsured, %	20.8 (16.3-25.3)	18.6 (13.7-24.6)	20.2 (16.0-24.9)	23.7 (20.5-26.4)	<.001
**Physical environment**					
RUCA code (micropolitan)	5.2 (2.4-8.0)	6.2 (2.5-9.4)	4.7 (2.0-7.6)	5.3 (3.4-7.5)	<.001
Violent crime rate per 100 000 population	199.0 (112.0-332.2)	161.5 (89.0-262.4)	200.4 (119.1-334.9)	272.8 (155.3-431.3)	<.001
Housing vacancy rate, %	10.6 (7.5-14.2)	8.3 (5.9-12.6)	10.3 (7.9-13.4)	13.7 (11.3-16.1)	<.001
No. of establishments in 2014	542.5 (225.0-1473.5)	609.5 (209.2-2162.2)	651.0 (261.0-1720.0)	380.0 (205.0-776.0)	<.001
Food desert, %	13.0 (0-28.6)	6.8 (0-22.0)	14.3 (0-26.9)	22.2 (0-35.7)	<.001
Access to exercise, %	62.1 (43.3-77.3)	68.7 (49.5-84.8)	63.5 (46.0-76.7)	48.3 (33.0-64.7)	<.001
**Health status and behaviors**					
BMI >30, %	31.2 (28.5-33.7)	28.6 (25.5-31.3)	31.4 (29.3-33.4)	34.4 (32.0-36.9)	<.001
T2DM, %	10.9 (9.6-12.5)	9.6 (8.6-10.9)	10.9 (9.9-12.1)	12.9 (11.7-14.3)	<.001
Current smoking, %	17.8 (15.7-20.7)	15.8 (14.6-17.1)	18.3 (16.2-20.3)	21.8 (19.6-23.9)	<.001
No leisure physical activity, %	27.7 (23.9-30.9)	23.8 (19.9-27.3)	27.9 (24.9-30.3)	32.2 (29.6-35.1)	<.001
Frequent distress, %					
Mental	11.1 (9.6-12.6)	9.6 (8.6-10.9)	11.2 (10.1-12.3)	13.2 (12.2-14.3)	<.001
Physical	11.2 (9.7-13.2)	9.7 (8.7-11.0)	11.2 (10.1-12.6)	13.9 (12.4-15.2)	<.001
Adults with fair/poor health, %	15.9 (13.0-20.1)	12.9 (11.5-15.0)	16.2 (13.9-19.1)	21.4 (18.8-24.1)	<.001

Abbreviations: BMI, body mass index (calculated as weight in kilograms divided by height in meters squared); RUCA, rural-urban commuting area; T2DM, type 2 diabetes.

^a^ Includes individuals self-identifying as Black or African American, American Indian or Alaska Native, Asian, Native Hawaiian, Pacific Islander, other, or multiple races/ethnicities.

^b^ Scores range from 0 to 100, with higher scores indicating higher levels of distress.

**Figure 3.**
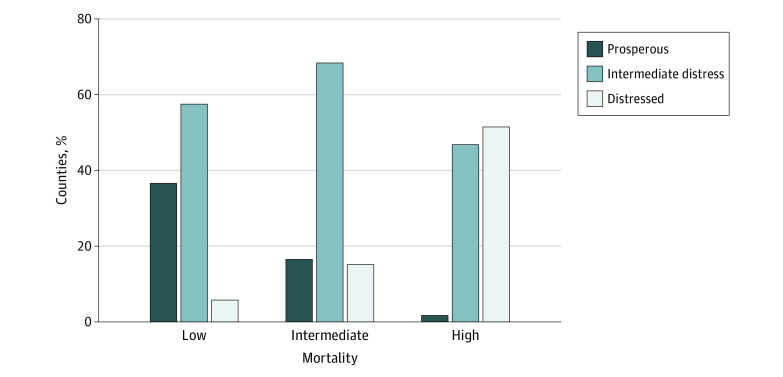
Proportion of Counties Qualified as Prosperous, Intermediate Distress, or Distressed by Mortality Cluster Socioeconomic distress is based on aggregate distress scores across 7 individual metrics in the Distressed Communities Index (DCI) and grouped into quintiles ranging from prosperous counties to distressed counties, with low distress scores indicating the least distressed counties. The intermediate distress category is composed of the 3 intermediate categories (comfortable, midtier, and at-risk) in the DCI. The proportion of prosperous counties declined from the low- to high-mortality clusters, whereas the proportion of distressed counties increased from the low- to high-mortality clusters. More than half of high-mortality counties qualified as distressed based on distress score.

Prosperous counties, based on DCI distress score, were most prevalent in the low-mortality cluster (384 counties [36.6%]) and declined in the intermediate- (228 counties [16.5%]) and high-mortality (12 counties [1.7%]) clusters. Most counties (357 [51.5%]) in the high-mortality cluster met criteria for the highest level of socioeconomic distress (Figure 3). Two groups of outliers by DCI were identified, including counties with low levels of socioeconomic distress belonging to the high-mortality trajectory (n = 133), and counties with high levels of socioeconomic distress belonging to the low-mortality trajectory (n = 184). When compared with other low-distress counties, those with high-mortality were demographically distinct, with a higher prevalence of racial minority group residents (11.6% [IQR, 4.8%-25.8%] vs 7.4% [IQR, 3.9%-14.7%]), and demonstrated higher levels of independent social (eg, less than a high school education, 15.8% [IQR, 12.5%-19.0%] vs 10.3% [IQR, 8.0%-13.0%]), community (eg, housing vacancy, 105% [IQR, 8.5%-12.7%] vs 8.2% [IQR, 6.1%-11.0%]), and health-related (eg, current smoking, 20.7% [IQR, 17.9%-22.7%] vs 16.4% [IQR, 15.0%-18.4%]) risk factors (eTable 3 in the Supplement). In contrast, high-distress, low-mortality counties exhibited significantly lower levels of socioeconomic and community risk factors—including lower violent crime rates (217.5 [IQR, 116.3-329.1] vs 274 [IQR, 149.1-451.0] per 100 000 population), lower rates of unemployment (32.7% [IQR, 27.4%-39.2%] vs 35.4% [IQR, 31.1%-43.9%]), and improved educational metrics (20.3% [IQR, 17.0%-23.5%] vs 22.5% [IQR, 19.2%-25.7%])—when compared with other high-distress counties and exhibited a lower prevalence of racial minority group residents (11.8% [IQR, 5.9%-25.8%] vs 26.4% [IQR, 7.6%-44.5%]) (eTable 4 in the Supplement).

### Factors Associated With County-Level CV Mortality Trajectory

We observed substantial collinearity between certain health-related risk factors for CV mortality and social risk factors, such as educational attainment (correlation coefficient, 0.7 with type 2 diabetes), median income (correlation coefficient, 0.7 with fair/poor health and mental distress), poverty rates (correlation coefficient, 0.8 with physical distress), and lack of health insurance (correlation coefficient, 0.7 with fair/poor health) (eTable 5 in the Supplement). In hierarchical modeling, population demographics accounted for 18% of the variance in the CV mortality trajectory. The addition of socioeconomic factors in model 2 substantially improved the model’s predictive ability (model 2, 46%) (Table 2). The addition of environmental characteristics (model 3) and health risk factors and behaviors (model 4) further improved the variance in CV mortality trajectory explained by the model to 54% and 60%, respectively.

**Table 2.** Hierarchical Modeling of County Characteristics With Model Performance

County characteristic	OR (95% CI)^a^							
	Model 1 (*R*^2^ = 0.18)		Model 2 (*R*^2^ = 0.46)		Model 3 (*R*^2^ = 0.54)		Model 4 (*R*^2^ = 0.60)	
	High (vs low)	Intermediate (vs low)	High (vs low)	Intermediate (vs low)	High (vs low)	Intermediate (vs low)	High (vs low)	Intermediate (vs low)
Population density, persons per square mile	0.95 (0.84-1.07)	0.96 (0.89-1.05)	1.3 (1.00-1.59)	1.14 (0.92-1.41)	1.15 (0.99-1.34)	1.03 (0.90-1.19)	1.31 (1.04-1.65)	1.14 (0.92-1.42)
Racial minority group, %^b^	2.9 (2.6-3.3)	1.5 (1.4-1.7)	1.92 (1.61-2.28)	1.35 (1.16-1.57)	1.56 (1.28-1.91)	1.18 (0.99–1.40)	1.70 (1.35-2.14)	1.24 (1.02-1.51)
Hispanic, %	0.33 (0.27-0.41)	0.77 (0.71-0.84)	0.13 (0.10-0.16)	0.29 (0.25-0.33)	0.09 (0.07-0.12)	0.23 (0.20-0.27)	0.23 (0.17-0.30)	0.39 (0.33-0.47)
Male, %	0.89 (0.80-0.99)	0.89 (0.82-0.97)	0.68 (0.60-0.78)	0.75 (0.68-0.83)	0.77 (0.66-0.89)	0.82 (0.73-0.92)	0.88 (0.75-1.03)	0.90 (0.79-1.01)
Less than high school education, %	NA	NA	13.58 (10.5-17.6)	6.5 (5.42-7.82)	14.93 (11.22-19.86)	6.97 (5.56-8.74)	6.17 (4.55-8.36)	3.70 (2.91-4.70)
Median household income, $	NA	NA	0.35 (0.27-0.46)	0.96 (0.85-1.08)	0.43 (0.32-0.58)	1.05 (0.91-1.20)	0.63 (0.45-0.87)	1.17 (1.01-1.36)
Food desert prevalence, %	NA	NA	NA	NA	0.82 (0.71-0.96)	0.91 (0.81-1.02)	0.89 (0.75-1.05)	0.95 (0.85–1.07)
Violent crime rate, %	NA	NA	NA	NA	1.80 (1.49-2.17)	1.48 (1.27-1.72)	1.58 (1.29-1.94)	1.38 (1.17-1.62)
Access to exercise opportunities, %	NA	NA	NA	NA	1.13 (0.95-1.36)	1.19 (1.04-1.36)	1.53 (1.26-1.87)	1.39 (1.20-1.60)
Housing vacancy rate, %	NA	NA	NA	NA	1.64 (1.37-1.96)	1.26 (1.11-1.42)	1.25 (1.03-1.53)	1.02 (0.88-1.17)
BMI >30	NA	NA	NA	NA	NA	NA	1.71 (1.31-2.21)	1.48 (1.25-1.76)
Smoking prevalence, %	NA	NA	NA	NA	NA	NA	2.04 (1.58-2.64)	1.56 (1.29-1.89)
No physical activity, %	NA	NA	NA	NA	NA	NA	3.74 (2.83-4.93)	1.88 (1.55-2.27)

Abbreviations: BMI, body mass index (calculated as weight in kilograms divided by height in meters squared); NA, not applicable; OR, odds ratio.

^a^ Sequential multinomial logistic regression models were fitted incorporating population and demographic characteristics (model 1), sociodemographic characteristics (model 2), environmental attributes (model 3), and health and behavioral characteristics (model 4). Associations between individual county attributes (per 1-SD higher value) and cardiovascular mortality trajectory are reported as ORs with 95% CIs, with low-mortality trajectory counties serving as the reference group for comparison.

^b^ Includes individuals self-identifying as Black or African American, American Indian or Alaska Native, Asian, Native Hawaiian, Pacific Islander, other, or multiple races/ethnicities.

In the fully adjusted model, higher population density and a greater proportion of racial minority group residents were associated with a significantly higher probability of the highest mortality cluster (OR, 1.31 [95% CI, 1.04-1.65] and OR, 1.70 [95% CI, 1.35-2.14], respectively; reference group, lowest-mortality trajectory) (Table 2). By contrast, a higher proportion of Hispanic residents (vs racial minority group residents) was associated with a significantly lower probability of highest mortality cluster trajectory (OR, 0.23 [95% CI, 0.17-0.30]). Among socioeconomic factors, lack of high school education was associated with a significantly higher probability of high-mortality trajectory (OR, 6.17 [95% CI, 4.55-8.36]), whereas median household income was inversely associated with a significantly higher likelihood of both the intermediate- (OR, 1.17 [95% CI, 1.01-1.36]) and high-mortality (OR, 0.63 [95% CI, 0.45-0.87]) trajectory clusters (Table 2). Among physical and environmental characteristics, a significant association with a higher likelihood of the high-mortality trajectory was found for higher violent crime rates (OR, 1.58 [95% CI, 1.29-1.94]), access to exercise opportunities (OR, 1.53 [95% CI, 1.26-1.87]), and housing vacancy rates (OR, 1.25 [95% CI, 1.03-1.53]). A high prevalence of obesity and high-risk health behaviors were significantly associated with high-mortality cluster membership and were associated with cluster membership in the high-mortality trajectory, including body mass index greater than 30 (OR, 1.71 [95% CI, 1.31-2.21]), smoking (OR, 2.04 [95% CI, 1.58-2.64]), and physical inactivity (OR, 3.74 [95% CI, 2.83-4.93]) (Table 2).

## Discussion

We observed several significant findings in our study. First, from 1980 to 2014, mortality declined substantially in the US by 45.5%. Second, we identified 3 distinct clusters of counties based on CV mortality trajectory during the study period, with the clusters differing significantly in their CV mortality rates throughout the study period but demonstrating a similar absolute decline in CV mortality. Third, county-level and regional disparities in CV mortality were unchanged and persisted across the county clusters for 35 years. Fourth, we found that social, environmental, and behavioral risk factors—namely, lower educational attainment, higher violent crime rates, higher smoking prevalence, and higher rates of physical inactivity—were associated with the highest CV mortality trajectory.

Consistent with our observation, prior studies have demonstrated a substantial decline in CV mortality since the 1960s.^5,6,12,13^ We further extend these observations by evaluating heterogeneity in the patterns of CV mortality decline and identified 3 distinct county-level trajectories of CV mortality with parallel declining trends in mortality over 35 years. Among all clusters, we observed a plateauing in CV mortality reductions during the most recent period from 2010 to 2014, with mortality rates among the high-mortality cluster increasing marginally. Notably, our study adds to existing observations by demonstrating that counties that reported the highest CV mortality levels in 1980 maintained high levels of CV mortality during the study period. Similarly, counties that reported the lowest CV mortality at onset maintained the lowest mortality rates during the follow-up period. As a result, gaps in performance between high- and low-mortality counties persisted throughout the 35 years, despite overall improvements in mortality rates, suggesting that reductions in mortality have not translated into reductions in CV disparities.

We observed a distinct geographic clustering of counties with different CV mortality trajectories. High-mortality counties were concentrated in the Deep South, whereas intermediate-mortality counties were located throughout the mid-Atlantic and Midwestern regions, and low-mortality counties were most widely distributed in the remaining US regions.^5,6,25,26^ These findings reflect the intersection between geography and demography: the Southeastern US has both the highest proportion of Black residents and counties with the lowest neighborhood socioeconomic status—2 key factors associated with the high-mortality trajectory identified in our study.

Our study also adds to the existing literature by providing a comprehensive assessment of the association of CV mortality trajectories with county-level social, environmental, and health-related risk factors. We observed that county traits across multiple domains, including socioeconomic factors (educational status and income), environmental factors (violent crime and housing vacancy rates), and health behaviors (smoking, obesity, and physical inactivity), were associated with the high-mortality trajectory. The importance of educational attainment, poverty, and CV risk factors has been demonstrated previously.^27,28,29,30,31,32^ However, our findings extend these observations to evaluate the intersections between these domains and the relative importance of community-level factors in contributing to population risk.

Traditional CV risk behaviors and risk factors have been the target of substantial public health investment over the past 4 decades and are likely responsible for much of the observed gains in national CV mortality reduction observed in this study.^33^ We identified health behaviors and risk factors such as physical inactivity and smoking to be significantly associated with CV mortality trajectory, highlighting the importance of progress made in this area.^33^ However, slowing in mortality declines across clusters and persistent gaps between clusters raise some concerns. First, traditional CV risk factors, including smoking prevalence, remain above target in many parts of the country.^34^ Second, other nontraditional risks, including structural and social factors, may be driving residual risk of disease, particularly among the most disadvantaged counties.

The contribution of structural and societal factors to the persisting county-level disparities in CV health is further corroborated by our observations of associations between upstream socioeconomic and societal factors such as educational attainment, income, and environment with the likelihood of a high-mortality trajectory. Low educational attainment was associated with a 6-fold higher likelihood of high CV mortality trajectory and was also associated with every adverse health-related risk covariate included in the study, highlighting the important overlap between educational attainment and poverty with traditional CV risk factors.^27,29^ Smaller, regional studies^35^ have also linked higher levels of violent crime to increases in blood pressure, CV hospital admissions, and missed medical appointments, demonstrating the community environment’s role in driving lifetime chronic disease risk. Recent studies^36^ have also shown ties between neighborhood social environment metrics and social cohesion, including violence rates and housing conditions, physical activity levels and obesity, and downstream CV risk. These relationships are highlighted by the existence of outlier counties, particularly socioeconomically prosperous counties with high CV mortality, which were characterized by violent crime rates that far exceeded low-mortality counterparts and disproportionally high levels of neighborhood deprivation represented by high housing vacancy rates, a high proportion of food deserts, and low levels of reported access to exercise opportunities.

Our study has several important implications for public health. We demonstrate that despite tremendously successful national initiatives to improve CV health, more than two-thirds of US counties remain on intermediate- or high-mortality trajectories. We also identify multiple county-level targets for investment to affect CV health moving forward. Specifically, investment in upstream social factors and health behavior factors may yield more lasting health gains and help close the gaps in performance across counties. For example, interventions targeted at increasing rates of home ownership and safety and transforming neighborhood design have demonstrated the potential to affect social vulnerability and achieve health gains by means of social investment and policy initiatives.^37,38^ Similarly, educational gains early in life achieved with early childhood educational interventions may translate to long-term reductions in unhealthy behaviors, violent crime, and adverse health outcomes.^39^ Moreover, a substantial body of evidence links reductions in CV disease to policy and behavioral interventions in the form of tobacco sales taxes, clean air legislation, and financial incentives tied to school and workplace physical activity.^40^ Such interventions in concert represent a new paradigm that our study suggests may be required to address social needs in high-risk communities and to close the gap between the highest and lowest CV mortality communities. More rigorous study aimed at evaluating the strengths of specific policies and interventions in a modern context will be needed to guide a long-term strategy for addressing CV health disparities.

### Limitations

Our study has some important limitations. First, as in prior county-level mortality analyses using GBD data,^5,6^ measurement of CV mortality depended on vital statistics and census population data collected locally and death certificate *International Classification of Disease* coding. It may be subject to missing or incorrectly allocated data, and the latter is subject to misclassification error.^41^ In addition, our county-level exposures assessment represents cross-sectional data from 2011 to 2014 only, and outcomes data reflect data to 2014 only, because this is the latest time point reported by the Institute for Health Metrics and Evaluation for county-level mortality. The approach to clustering in the present analysis represents a simple and easily interpretable clustering strategy selected to assess county-level factors associated with high-mortality trajectories. Future studies are needed to compare other approaches of clustering, including those using a spatiotemporal Gaussian process, and to determine the most optimal approach for county-level clustering. Our analysis does not reflect changes in county characteristics owing to a lack of existing data from some of our data sets before 2010. Future efforts to capture changes in social exposures over time will be valuable in further informing how changes in social factors have correlated with outcomes over time. Finally, the present analysis reports population-level data; therefore, direct inferences about individual-level risk and behavior cannot be made. Our approach does not capture differences in policy implementation and governance across counties and thus cannot inform causality regarding interventions that may have affected CV mortality on a county or a regional level during the 35-year study period. More granular assessments of community-level risks and interventions will allow for a better understanding of how actions at the community level interact and may affect regional disparities moving forward.

## Conclusions

Despite improvements in CV mortality in the US in recent decades, considerable disparities remain at the county level between high- and low-mortality counties. We found no change in the mortality gap between high- and low-mortality counties during our 35-year study period. High-mortality trajectory counties were further characterized by significant social dysfunction and an excess burden of CV risk factors, including lower educational attainment, higher levels of violent crime, and higher prevalence of smoking and physical inactivity.

## Notes

Supplement. **eMethods.** Data Sources **eTable 1.** Data Sources for Relevant County-Level Exposure Variables **eTable 2.** Annual Rate of Decline by Generalized Linear Mixed-Effects Modeling of CV Mortality for All US Counties and by Trajectory Cluster, 1980-2014 **eTable 3.** Characteristics of Outlier Counties Comparing Low-Distress Counties Belonging to the High Mortality Trajectory vs Those With Low or Intermediate Mortality **eTable 4.** Characteristics of Outlier Counties Comparing High-Distress Counties Belonging to the Low-Mortality Trajectory vs Those With High or Intermediate Mortality **eTable 5.** Pairwise Pearson Correlation Coefficients Among County-Level Exposure Levels
