# Supplementary material for: Longitudinal Trajectories and Factors Associated With US County-Level Cardiovascular Mortality, 1980 to 2014
Source: JAMA Netw Open. 2021 Nov 30;4(11):e2136022. doi: 10.1001/jamanetworkopen.2021.36022 (PMC8634057; doi:10.1001/jamanetworkopen.2021.36022)
Supplement: Supplement. — eMethods. Data Sources eTable 1. Data Sources for Relevant County-Level Exposure Variables eTable 2. Annual Rate of Decline by Generalized Linear Mixed-Effects Modeling of CV Mortality for All US Counties and by Trajectory Cluster, 1980-2014 eTable 3. Characteristics of Outlier Counties Comparing Low-Distress Counties Belonging to the High Mortality Trajectory vs Those With Low or Intermediate Mortality eTable 4. Characteristics of Outlier Counties Comparing High-Distress Counties Belonging to the Low-Mortality Trajectory vs Those With High or Intermediate Mortality eTable 5. Pairwise Pearson Correlation Coefficients Among County-Level Exposure Levels [file jamanetwopen-e2136022-s001.pdf]

Supplementary Online Content

Rao S, Hughes A, Segar MW, et al. Longitudinal trajectories and factors associated with US county-level cardiovascular mortality, 1980 to 2014. *JAMA Netw Open*. 2021;4(11):e2136022. doi:10.1001/jamanetworkopen.2021.36022

eMethods. Data Sources

eTable 1. Data Sources for Relevant County-Level Exposure Variables

eTable 2. Annual Rate of Decline by Generalized Linear Mixed-Effects Modeling of CV Mortality for All US Counties and by Trajectory Cluster, 1980-2014

eTable 3. Characteristics of Outlier Counties Comparing Low-Distress Counties Belonging to the High Mortality Trajectory vs Those With Low or Intermediate Mortality

eTable 4. Characteristics of Outlier Counties Comparing High-Distress Counties Belonging to the Low-Mortality Trajectory vs Those With High or Intermediate Mortality

eTable 5. Pairwise Pearson Correlation Coefficients Among County-Level Exposure Levels

This supplementary material has been provided by the authors to give readers additional information about their work.

© 2021 Rao S et al. *JAMA Network Open*.

eMethods. *Data Sources*

Population characteristics and sociodemographic data were measured using 5-year estimates for 2011-2015 from the American Community Survey (ACS)[12], which administers surveys to approximately 295,000 households per month and aggregates results to the block group-, tract-, county-, and state-levels over 1 and 5 years.[13] We included measures of counties' racial and ethnic composition, gender distribution, proportion of the population over the age of 55, income, education and violent crime rates. Economic distress, measured by housing vacancy rate, proportion of adults not in work, proportion of population in a distressed zip code and aggregate distress score, was reported based on data collected from the Distressed Communities Index (DCI) using data from ACS 5-year estimates and Census Bureau Business Patterns datasets.[14] The DCI additionally compiles an aggregate score, termed the distress score, for each county accounting for performance across seven individual metrics (< high-school education, housing vacancy rate, adults not in work, poverty rate, median income ratio, and change in employment and establishment proportions from 2014-2018). Scores range from 0-100, reflecting how prosperous (low score) or distressed (high score) a county is. Counties are then grouped by score into quintiles that reflect whether a community is prosperous, comfortable, mid-tier, at-risk, or distressed. Population distribution was determined from the United States Department of Agriculture (USDA) Economic Research Service (ERS) Rural-Urban Continuum Codes (RUCC) codes, which classifies U.S. counties as metropolitan versus non-metropolitan based on total population adjacent to a metropolitan area, percentage of labor force commuting to metropolitan counties, and population density.[12,15] The health status of

© 2021 Rao S et al. *JAMA Network Open*.

the population was measured using data reported in the 2014 County Health Rankings dataset[16] which captures county-level data on prevalence of health outcomes, risk factors and physical environment including prevalence of obesity, diabetes, smoking, and self-reported metrics of fair or poor health. A summary measure of the food environment in each county and the percent of tracts within a county classified as a food desert in 2010 was downloaded from the USDA's Food Environment Atlas.[17] The USDA classifies tracts as being in a food desert if at least 500 people or one-third of the population lives greater than a threshold distance (1 mile in an urban area; 10 in a rural area) from a large grocery store. A list of included covariates and data sources is provided in **eTable 1**.

© 2021 Rao S et al. *JAMA Network Open*.

eTable 1. Data Sources for Relevant County-Level Exposure Variables

**Population characteristics** *(2011-2014 ACS 5-year estimates)*	**Population Distribution** *(USDA Economic Research Service (ERS) Rural-Urban Continuum Codes dataset)*	**Distressed Communities Index** *(2014-2018 ACS 5-year estimates and Census Bureau Business Patterns datasets)*	**Population Health Status** *(2014 CHR dataset)*	**County Food Environment** *(USDA Food Environmental Atlas)*
Non-white (%)	RUCA Code	Distress Score	BMI > 30	Proportion of tracts within a county that qualify as a food dessert
Hispanic (%)		Housing Vacancy Rate	Diabetes (%)	
Male (%)		Change in employment (%)	Currently smoking (%)	
Age >= 55 (%)		Change in establishments (%)	Fair/poor health (%)	
Median household income		Population in distressed zip codes (%)	Mentally unhealthy days/month	
< HS education (%)		Adults not in work (%)	No leisure-time physical activity	
Violent crime rate		Housing Vacancy Rate	Access to exercise (%)	
		Change in employment (%)	Frequent mental distress (%)	
		Change in establishments (%)	Frequent physical distress (%)	
			Uninsured (%)	

© 2021 Rao S et al. *JAMA Network Open*.

eTable 2. Annual Rate of Decline by Generalized Linear Mixed-Effects Modeling of CV Mortality for All US Counties and by Trajectory Cluster, 1980-2014

Time Period	***Rate of change in CV mortality in county-level clusters***		
	Low-mortality cluster	***Intermediate-mortality cluster***	***High-mortality cluster***
(1980-1990)	*-0.73 (-0.73, -0.72)*	*-0.77 (-0.77, -0.78)*	*-0.70 (-0.70, -0.69)*
(1990-1999)	*-0.69 (-0.70, -0.69)*	*-0.74 (-0.75, -0.74)*	*-0.67 (-0.67, -0.66)*
(2000-2009)	*-0.85 (-0.85, -0.84)*	*-1.00 (-1.00, -0.99)*	*-1.07 (-1.07, -1.06)*
(2010-2014)	*-0.06 (-0.07, -0.05)*	*-0.04 (-0.05, -0.04)*	*0.013 (0.001, 0.03)*

© 2021 Rao S et al. *JAMA Network Open*.

eTable 3. Characteristics of Outlier Counties Comparing Low-Distress Counties Belonging to the High Mortality Trajectory vs Those With Low or Intermediate Mortality

Outlier counties with low distress and high mortality differed from other low-distress counties due to an excess burden of social, community, and health-related risk factors as well as demographic differences including a higher proportion of non-White residents.

		Low Distress		
**Characteristic**		**High-mortality (n = 133)**	**Intermediate- or low-mortality (n = 1691)**	**P-value**
**Demographics**	Population density	68.1 [37.9, 130.3]	51.9 [14.5, 167.5]	0.012
	Male (%)	49.1 [48.6, 50.1]	49.7 [49.0, 50.4]	<0.001
	Age > 55 (%)	0.3 [0.3, 0.3]	0.3 [0.3, 0.4]	0.004
	Non-White (%)	11.6 [4.8, 25.8]	7.4 [3.9, 14.7]	<0.001
	Hispanic (%)	2.6 [1.6, 4.9]	4.1 [2.1, 9.2]	<0.001
	Foreign-born population (%)	1.9 [1.2, 3.3]	3.2 [1.7, 6.8]	<0.001
**Socioeconomic metrics**	Distress Score (0-100)	48.0 [36.9, 59.4]	28.5 [13.9, 45.6]	<0.001
	Population in Distressed Zip Code (%)	2.0 [0.0, 8.0]	0.0 [0.0, 2.0]	<0.001
	Median household income ($)	43K [40K, 47K]	51K [45K, 58K]	<0.001
	Poverty Rate (%)	15.5 [13.1, 18.0]	11.7 [9.2, 14.4]	<0.001
	< HS education (%)	15.8 [12.5, 19.0]	10.3 [8.0, 13.0]	<0.001
	Adults not in work (%)	25.7 [23.3, 29.2]	19.7 [16.3, 23.5]	<0.001
	Uninsured (%)	21.3 [18.9, 24.2]	17.7 [13.9, 22.3]	<0.001
**Physical environment**	RUCA Code	4.3 [2.0, 6.6]	4.9 [2.0, 8.3]	0.047
	Violent crime rate (per 100,000 population)	262.8 [156.3, 393.3]	165.8 [96.1, 262.4]	<0.001
	Housing Vacancy Rate (%)	10.5 [8.5, 12.7]	8.2 [6.1, 11.0]	<0.001
	Establishments in 2014 (n)	511.0 [265.0, 967.0]	775.0 [295.0, 2269.5]	0.001
	Food desert (%)	17.6 [0.0, 30.0]	7.7 [0.0, 20.0]	<0.001
	Access to exercise (%)	58.5 [41.4, 69.3]	68.2 [52.9, 82.5]	<0.001
**Health status & behaviors**	BMI > 30 (%)	32.8 [30.9, 34.7]	29.9 [27.1, 32.2]	<0.001
	T2DM (%)	11.6 [10.5, 12.5]	9.9 [8.9, 10.9]	<0.001
	Current smoking (%)	20.7 [17.9, 22.7]	16.4 [15.0, 18.4]	<0.001
	No leisure physical activity (%)	31.0 [28.0, 33.8]	25.3 [21.9, 28.4]	<0.001
	Frequent mental distress (%)	12.2 [11.4, 13.5]	10.1 [8.9, 11.3]	<0.001
	Frequent physical distress (%)	12.5 [11.2, 13.9]	10.0 [8.9, 11.2]	<0.001

© 2021 Rao S et al. *JAMA Network Open*.

	Adults with fair/poor health (%)	18.6 [16.3, 21.2]	13.5 [11.9, 15.6]	<0.001

© 2021 Rao S et al. *JAMA Network Open*.

eTable 4. Characteristics of Outlier Counties Comparing High-Distress Counties Belonging to the Low-Mortality Trajectory vs Those With High or Intermediate Mortality

Outlier counties with high distress but low mortality exhibited a high burden of health risk factors, but were demographically distinct from other high-distress counties with a significantly lower proportion of non-White residents, and significantly lower levels of socioeconomic and community risk factors.

		**High Distress**		
**Characteristic**		**Low mortality (n = 184)**	**Intermediate or High-mortality (n = 350)**	**P-value**
**Demographics**	Population density	27.0 [11.7, 47.1]	30.8 [15.7, 46.5]	0.44
	Male (%)	49.5 [48.8, 51.6]	49.4 [48.6, 51.2]	0.141
	Age > 55 (%)	0.3 [0.3, 0.3]	0.3 [0.3, 0.3]	0.001
	Non-White (%)	11.8 [5.9, 25.8]	26.4 [7.6, 44.5]	<0.001
	Hispanic (%)	3.1 [1.6, 14.6]	2.7 [1.4, 7.6]	0.072
	Foreign-born population (%)	2.2 [0.9, 4.6]	1.6 [0.9, 3.7]	0.096
**Socioeconomic metrics**	Distress Score (0-100)	86.8 [78.2, 93.5]	90.1 [82.8, 96.2]	<0.001
	Population in Distressed Zip Code (%)	100.0 [89.0, 100.0]	100.0 [91.0, 100.0]	0.174
	Median household income ($)	38K [34K, 41K]	34K [31K, 37K]	<0.001
	Poverty Rate (%)	19.9 [16.6, 23.6]	23.6 [20.2, 27.8]	<0.001
	< HS education (%)	20.3 [17.0, 23.5]	22.5 [19.2, 25.7]	<0.001
	Adults not in work (%)	32.7 [27.4, 39.2]	35.4 [31.1, 43.9]	<0.001
	Uninsured (%)	24.9 [22.1, 29.4]	25.5 [22.5, 28.2]	0.771
**Physical environment**	RUCA Code	7.0 [4.4, 9.0]	6.7 [4.9, 8.0]	0.377
	Violent crime rate (per 100,000 population)	217.5 [116.3, 329.1]	274.1 [149.1, 451.0]	0.001
	Housing Vacancy Rate (%)	15.0 [12.4, 17.6]	15.2 [13.2, 17.6]	0.254
	Establishments in 2014 (n)	235.0 [121.0, 483.5]	248.5 [142.0, 438.5]	0.522
	Food desert (%)	25.0 [0.0, 46.6]	27.0 [0.0, 50.0]	0.486
	Access to exercise (%)	50.6 [30.7, 66.8]	43.3 [29.0, 57.5]	0.004
**Health status & behaviors**	BMI > 30 (%)	32.2 [30.1, 34.4]	34.3 [31.1, 37.7]	<0.001
	T2DM (%)	12.7 [11.6, 13.9]	13.8 [12.5, 15.3]	<0.001
	Current smoking (%)	20.1 [16.9, 22.4]	22.5 [19.2, 25.0]	<0.001
	No leisure physical activity (%)	29.8 [28.0, 32.9]	32.0 [29.2, 35.2]	<0.001
	Frequent mental distress (%)	12.4 [10.8, 13.4]	13.7 [12.5, 14.7]	<0.001
	Frequent physical distress (%)	13.1 [11.7, 14.7]	14.7 [13.2, 16.4]	<0.001

© 2021 Rao S et al. *JAMA Network Open*.

	Adults with fair/poor health (%)	19.9 [17.9, 22.9]	23.4 [20.2, 26.5]	<0.001

© 2021 Rao S et al. *JAMA Network Open*.

eTable 5. Pairwise Pearson Correlation Coefficients Among County-Level Exposure Levels

Where two variables demonstrated a correlation of 0.6 or greater, the variable believed to be more upstream in the causal pathway was retained and the downstream variable excluded.

	County-level Chracterstics	1	2	3	4	5	6	7	8	9	10	11	12	13	14	15	16	17	18	19	20	21	22	23	24	25	26
1	Population density	**1**																									
2	Non-white (%)	0.3	**1**																								
3	Hispanic (%)	0.1	0.4	**1**																							
4	Male (%)	0.4	0.2	0.1	**1**																						
5	> 55 years (%)	0.5	0.5	0.4	0	**1**																					
6	Median Income	0.3	0.1	0.2	0.1	0.2	**1**																				
7	< HS education (%)	0.1	0.3	0.1	0	0.1	0.7	**1**																			
8	Uninsured (%)	0.2	0.4	0.3	0	0.1	0.5	0.7	**1**																		
9	Violent crime rates	0.3	0.5	0.3	0.2	0.3	0.2	0.3	0.3	**1**																	
10	Access to exercise opportunities (%)	0.6	0.1	0.3	0.2	0.3	0.4	0.3	0.2	0.2	**1**																
11	Food desert (%)	0	0.3	0.1	0.1	0.1	0.4	0.3	0.3	0.3	0.1	**1**															
12	Mean RUCA*	0.7	0.3	0.2	0.3	0.5	0.3	0.1	0.1	0.4	0.5	0.1	**1**														
13	BMI>30	0.1	0.1	0.3	0.1	0	0.5	0.4	0.2	0.1	0.4	0.2	0.1	**1**													
14	DM (%) *	0.2	0.3	0.2	0.2	0.3	0.8	0.7	0.6	0.2	0.4	0.3	0.2	0.5	**1**												
15	Smoking (%)	0.1	0.2	0.4	0.1	0.1	0.6	0.5	0.3	0.2	0.3	0.3	0	0.6	0.6	**1**											
16	Poor/fair health (%)	0.0	0.4	0	0.2	0.2	0.7	0.8	0.7	0.4	0.3	0.4	0.1	0.5	0.8	0.7	**1**										
17	No leisure time activity (%)	0.2	0	0.3	0.1	0.1	0.6	0.5	0.3	0	0.5	0.1	0.2	0.7	0.6	0.6	0.6	**1**									
18	Average # mentally unhealthy days	0.2	0.3	0.2	0.3	0.1	0.7	0.6	0.4	0.3	0.2	0.3	0.1	0.5	0.6	0.8	0.8	0.4	**1**								
19	Frequent mental distress	0.2	0.3	0.2	0.3	0.1	0.7	0.6	0.5	0.3	0.2	0.4	0.1	0.5	0.7	0.8	0.9	0.5	1	**1**							
20	Frequent physical distress*	0	0.4	0.1	0.2	0.1	0.8	0.7	0.6	0.4	0.3	0.4	0	0.5	0.8	0.8	1	0.5	0.9	1	**1**						

© 2021 Rao S et al. *JAMA Network Open*.

**21**	**Foreign-born (%)**	0.3	0.4	0.8	0	0.4	0.4	0	0.2	0.3	0.5	0	0.4	0.4	0.3	0.4	0.1	0.5	0.2	0.2	0.2	**1**					
**22**	**Population in distressed zip code (%)**	0.1	0.3	0	0.1	0	0.6	0.6	0.5	0.3	0.3	0.3	0.1	0.4	0.6	0.5	0.7	0.5	0.5	0.6	0.6	0.2	**1**				
**23**	**Poverty rate (%)**	0	0.4	0.1	0.1	0.1	0.8	0.7	0.6	0.3	0.2	0.4	0	0.4	0.7	0.6	0.8	0.4	0.7	0.8	0.8	0.2	0.7	**1**			
**24**	**Adults not in work (%)**	0.1	0.3	0	0.1	0	0.7	0.7	0.5	0.3	0.3	0.3	0.1	0.4	0.6	0.6	0.7	0.4	0.7	0.7	0.8	0.2	0.7	0.7	**1**		
**25**	**Housing vacancy rate (%)**	0.5	0.1	0.1	0	0.2	0.6	0.5	0.5	0.1	0.5	0.2	0.4	0.4	0.6	0.4	0.5	0.6	0.3	0.3	0.5	0.3	0.6	0.5	0.5	**1**	
**26**	**Establishments in 2014 (n)**	0.8	0.3	0.3	0.3	0.4	0.4	0.3	0.3	0.4	0.7	0.1	0.7	0.3	0.4	0.1	0.2	0.4	0	0	0.1	0.5	0.2	0.2	0.2	0.6	**1**

© 2021 Rao S et al. *JAMA Network Open*.
